# Improving Thermostability and Catalytic Activity of Glycosyltransferase From *Panax ginseng* by Semi-Rational Design for Rebaudioside D Synthesis

**DOI:** 10.3389/fbioe.2022.884898

**Published:** 2022-04-27

**Authors:** Meiqi Chen, Fangwei Song, Yuxi Qin, Shuangyan Han, Yijian Rao, Shuli Liang, Ying Lin

**Affiliations:** ^1^ Guangdong Key Laboratory of Fermentation and Enzyme Engineering, School of Biology and Biological Engineering, South China University of Technology, Guangzhou, China; ^2^ Key Laboratory of Carbohydrate Chemistry and Biotechnology, Ministry of Education, School of Biotechnology, Jiangnan University, Wuxi, China

**Keywords:** glycosyltransferase, rebaudioside D, semi-rational design, thermostability, enzyme activity

## Abstract

As a natural sweetener and sucrose substitute, the biosynthesis and application of steviol glycosides containing the component rebaudioside D have attracted worldwide attention. Here, a glycosyltransferase PgUGT from *Panax ginseng* was first reported for the biosynthesis of rebaudioside D. With the three-dimensional structures built by homology modeling and deep-learning–based modeling, PgUGT was semi-rationally designed by FireProt. After detecting 16 site-directed variants, eight of them were combined in a mutant Mut8 with both improved enzyme activity and thermostability. The enzyme activity of Mut8 was 3.2-fold higher than that of the wild type, with an increased optimum reaction temperature from 35 to 40°C. The activity of this mutant remained over 93% when incubated at 35°C for 2 h, which was 2.42 times higher than that of the wild type. Meanwhile, when the enzymes were incubated at 40°C, where the wild type was completely inactivated after 1 h, the residual activity of Mut8 retained 59.0% after 2 h. This study would provide a novel glycosyltransferase with great potential for the industrial production of rebaudioside D and other steviol glycosides.

## Introduction

Sweetness is an indispensable component of modern day life. Considering the health concerns caused by excessive intake of sucrose, the present trend of the prevailing market indicates that natural sucrose alternatives were appealing to consumers (Hagger et al., 2017; Archer, 2018; Mora and Dando, 2021). As one of the most widely used natural sweeteners, steviol glycosides (SGs) were low-calorie and zero glycemic indexes (Ceunen and Geuns, 2013; Castro-Munoz et al., 2022). SGs present positive effects on human health, including the improvement of metabolic health, assistance in weight control, and the benefits of lowering blood glucose, which attract the interest of many companies (Castro-Munoz et al., 2022). Excellent thermal (up to 80°C) and pH (2–10) stability allows them to be applied in the food and beverage industries (Kroyer, 2010). SGs contain a variety of sweet components, the highest levels of which are steviosides (St), followed by rebaudioside A (Reb A). The price of SG components is directly related to the nature and difficulty of extraction. Presently, with St as the substrate, a high yield of valuable Reb A was efficiently synthesized *via* biocatalysts (Chen et al., 2021). Among the SGs, rebaudioside D (Reb D) is one of the most important components, which has a much better flavor and higher sweetness than most other SGs, including Reb A and St (Kovacevic et al., 2018). However, the low content (0.42–0.5% w/w) of Reb D in the dry leaves of *Stevia rebaudiana* (*S. rebaudiana*) leads to difficulties in extraction and isolation, resulting in a high price (Hanson, 2016).

**SCHEME 1:**
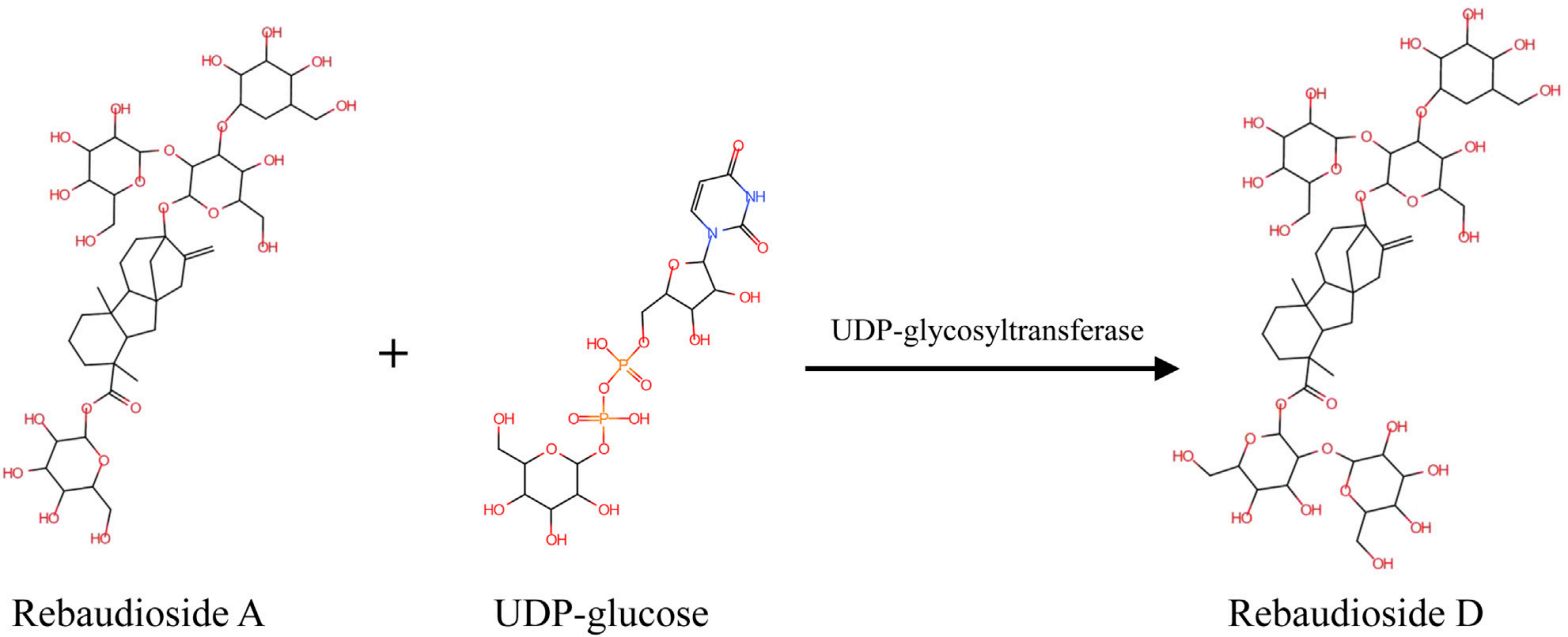
Mechanism of UGT-mediated synthesis of Reb D with Reb A and UDP-glucose as the substrates.

In a previous study, Reb D could be synthesized with Reb A as the substrate (Mohamed et al., 2011; Olsson et al., 2016). Glucose from UDP-glucose was transferred by specific β (1–2) glycosylation to the glucose on the C19-carboxylate of Reb A by UDP-glycosyltransferases (UGTs) (Zhang et al., 2021). Three UGTs were found to act as catalysts in the production of Reb D from Reb A (Scheme 1). The UGT91D2 from *S. rebaudiana* was the original enzyme for Reb D synthesis (Olsson et al., 2016). EUGT11 from *Oryza sativa* was heterologously expressed in *Pichia pastoris* to provide a whole-cell biocatalyst technology (Wang et al., 2020). Meanwhile, a computational strategy was developed to promote the activity of EUGT11 (Lin et al., 2020). In addition, the UGTSL2 from *Solanum lycopersicum* was applied for Reb D synthesis by expressing both in yeast and *Escherichia coli*, along with RM2 as a side product (Prakash et al., 2014; Chen et al., 2018). To promote the product specificity of UGTSL2, a mutant of UGSL2 was constructed as well (Chen L. et al., 2020). The synthesis of Reb D with enzymatic techniques was more efficient and environmentally friendly for the industry.

Nowadays, a biocatalyst is applied for the synthesis of complex compounds in a variety of fields. However, natural enzymes, without evolving for industrial environments, were often challenging for application (Foo et al., 2012). For biocatalytic reactions, the properties of enzymes play an important role in the cost and process of the entire industrial production process (Kirk et al., 2002). To improve the low activity and instability of enzymes, protein engineering was developed as a powerful tool to modify the structure (Xiong et al., 2021). The semi-rational design strategy combined the benefits of directed evolution and rational design (Chica et al., 2005; Sheldon and Pereira, 2017). Two distinct methodologies were involved in the semi-rational design: sequence-based enzyme redesign and structure-based enzyme redesign (Xiong et al., 2021). Proteins without a crystal structure or high-throughput determination methods could be redesigned for higher activity and better thermal stability by semi-rational design strategies (Nakano et al., 2018; Cheng et al., 2020).

In this study, a UDP-glycosyltransferase PgUGT from *Panax ginseng* (*P. ginseng*) catalyzing the bioconversion of Reb A to Reb D was first reported. The three-dimensional structure of PgUGT was modeled and redesigned by a semi-rational design strategy. Consequently, a mutant Mut8 containing eight altered residues was constructed with greater enzyme activity and enhanced thermostability after confirming the site-directed mutagenesis of selective noncatalytic residues.

## Materials and Methods

### Chemicals and Reagents

All reagent grade chemicals were purchased from Sigma-Aldrich (Steinheim, Germany). Restriction enzymes, DNA polymerase, and T4 DNA ligase were purchased from Thermo Scientific (United States). Standard samples of Reb A (97%) and Reb D (97%) were purchased from Qingdao Runde Biotechnology Company.

### Strains, Plasmids, and Culture Conditions

*E. coli* Top10 was used as the host strain for plasmid storage. The strain used for protein expression was *E. coli* BL21 (DE3) with pET30a (Invitrogen, Waltham, MA, United States) as vectors. The genes encoding EUGT11 (XP_015629141.1), LsUGT1 (XP_023735445.1), CsUGT1 (ALO19883.1), HaUGT1 (XP_022009959.1), AsUGT1 (AZQ26909.1), AtUGT1 (XP_020148974.1), PgUGT (A0A0A6ZFY4.1), ZmUGT1 (NP_001150595.1), EUGT11 (XP_015629141.1), UGTSL2 (XP_004250485.1), and UGT94-28-3 (Itkin et al., 2016) were retrieved from the NCBI database and articles, synthesized by Generay (Shanghai Generay Biotech Co. Ltd, China). The gene sequences were constructed into the pET30a vector.

The bacteria were cultivated in Luria–Bertani (LB) medium (1% NaCl, 0.5% yeast extract, and 1% tryptone) containing 50 μg/ml kanamycin in a shaking incubator.

### Cell-Free Protein Synthesis System for Protein Production

The cell extract of *E. coli* BL21 (DE3) was prepared and quantified using the reported protocol (Karim and Jewett, 2016).

Cell-free protein synthesis (CFPS) was carried out in a 1.5-ml centrifuge tube containing 60 μl volumes at 37°C for 16 h. Each reaction consisted of ATP (1.8 mM), GTP (1.3 mM), UTP (1.3 mM), CTP (1.3 mM), nicotinamide adenine dinucleotide (NAD; 0.4 mM), phosphoenolpyruvate (PEP; 33 mM), coenzyme-A (0.27 mM), putrescine (1 mM), spermidine (1.5 mM), HEPES (57 mM), folinic acid (0.1 mM), E. coli tRNA mixture (0.26 mg/ml), oxalic acid (4 mM), potassium glutamate (130 mM), magnesium glutamate (10 mM), 20 amino acids (2 mM each), and cell extracts (10 μl). For each reaction, 16 ng/μl plasmid was added. The soluble proteins were collected after centrifugation at 10,000 rpm for 5 min.

### Characteristics of Cell-Free Synthesis of Glycosyltransferase

The abilities of the proteins produced *via* CFPS were analyzed using Reb A as the substrate. A 200-μl reaction system in a 2-ml centrifuge tube was incubated at 35°C and 250 rpm for 12 h. The reaction solution contained 50 mM PBS (pH 8.0), 2 mM Reb A, 2 mM UDP-glucose, and 50 μl of precipitated proteins collected from the CFPS reaction. After incubation, 200 μl of phosphoric acid (0.9 M) was added to suspend the reaction and 200 μl of sodium hydrate (2 M) was added after 5 min to neutralize the reaction system. The reactants were determined by high-performance liquid chromatography (HPLC) to confirm the types of SGs.

### Heterologous Expression and Purification of PgUGT in *E. coli*

The plasmid pET30a-PgUGT was transformed to *E. coli* BL21 (DE3). The positive clones of BL21 (DE3)/PgUGT were verified and precultured in the LB medium containing 50 μg/ml kanamycin at 37°C overnight. The precultured cells were then transferred into a 100-ml LB medium with 50 μg/ml kanamycin and 0.5 mM isopropyl-β-D-thiogalactopyranoside (IPTG) was added to induce protein expression after the OD_600_ of the cells was in the range of 0.6–0.8. After 26 h of induction at 16°C, the cells were harvested by centrifugation at 7,000 rpm for 3 min at 4°C. The cells were washed twice with PBS buffer (pH 7.5) and disrupted by ultrasonication. The supernatant was collected by centrifugation at 10,000 rpm for 30 min at 4°C to obtain the crude enzyme.

Mutations of PgUGT were generated by site-directed mutagenesis by PCR based on the plasmid pET30a-PgUGT, and the primers are listed in Supplementary Table S1. After confirming by DNA-sequencing, the mutated plasmids were transformed into *E. coli* BL21 (DE3) for fermentation and induction as mentioned previously.

Purification of the enzymes was performed by 5-ml nickel affinity chromatography using Ni-NTA agarose (Novagen, United States). The mobile phase A was 20 mM sodium phosphate buffer (pH 7.5) adding 500 mM NaCl, and the mobile phase B was 20 mM sodium phosphate buffer (pH7.5) adding 500 mM NaCl and 250 mM imidazole. The non-target proteins were washed off with 80% phase A and 20% phase B. The target protein was eluted with 20% phase A and 80% phase B. The elution proteins were rebuffered to phase A with a 5-ml HiTrap Desalting column (Cytiva, United States). The purified protein was confirmed by sodium dodecyl sulfate-polyacrylamide gel electrophoresis (SDS-PAGE).

### Enzyme Activity Assay and Kinetic Parameters of PgUGT and Variants

The enzyme activities were carried out in a mixture (200 μl) of 5 mM Reb A and 2 mM UDP-glucose in 50 mM sodium phosphate buffer (pH 7.5) containing 0.02 mg/ml of the purified enzyme and incubated at 35°C for 10 min. After incubation, 200 μl phosphoric acid (0.9 M) was added to suspend the reaction and 200 μL of sodium hydrate (2 M) was added after 5 min to neutralize the reaction system. The reactants were determined by HPLC to confirm the concentrations of SGs. One unit (U) of glycosyltransferase activity was defined as the amount of enzyme that produced 1 μmol of Reb D from Reb A per minute under the described conditions.

The reactant containing 2 mM UDP-glucose, 0.1–1.8 mM Reb A, and 0.02 mg/ml purified enzyme in 50 mM sodium phosphate buffer (pH 7.5) was incubated at 35°C for 10 min. The suspending method of the reaction was the same as mentioned previously. The production of Reb D was determined by HPLC. The kinetic parameters (*k*_cat_, *K*_m_, and *k*_cat_/*K*_m_) were obtained by fitting data to the Michaelis–Menten equation by OriginPro 2018. Three replicate experiments were performed for each reaction.

### Temperature and pH Dependence of PgUGT and Mutants

The enzyme activity was measured at 25–50°C to reveal the optimal temperature of the enzymes with the pH of the reactant being 7.5. After incubation at different temperatures for 30 min, the residual enzyme activity was determined, indicating the thermostability of enzymes.

The optimal pH of PgUGT was determined at 35°C by measuring the activity in the range of 6.5–9.0. The pH stability was determined by measuring the residual enzyme activity at 35°C after incubation in the following buffers with different pH at 4°C for 24 h: 50 mM sodium phosphate buffer (6.5–8.0) and 50 mM Tris-HCl buffer (pH 8.0–9.5). Three replicate experiments were performed for each reaction.

### Structure Modeling and Semi-Rational Design

The three-dimensional structure of PgUGT was built in two ways. The homology model was obtained by Discovery Studio 2020 based on the crystal structure of PDB code 2vce, 5u6m, 5u6n, 5v2k, 6inf, 6ing, 6kvi, and 6o86. The other one was built by the online RoseTTAFold service (https://robetta.bakerlab.org/) (Baek et al., 2021). The models retrieved from different methods were evaluated with the scoring program SAVES v6.0 (https://saves.mbi.ucla.edu). The model with the highest scores was used as the input file for the FireProt online program (https://loschmidt.chemi.muni.cz/fireprot/) (Musil et al., 2017). Based on the predicted change in Gibbs free energy, FireProt provided two mutation lists after energy- and evolution-based calculation. Candidate mutants were selected from the given lists.

### HPLC Analysis

The reactants were centrifuged and the supernatant was filtered through a cellulose nitrate membrane (0.22 μm) for detection. The samples were analyzed by using a Waters 2,690–2489 HPLC system.

Steviol glycosides were detected by UV detection at 210 nm. An HPLC analysis was performed using a Sepax Sapphire-C18 5 column (250 mm × 4.6 mm) at 40°C. The flow rate was set to 1 ml/min with 68% mobile phase A (1.38 g/L sodium phosphate buffer, pH 2.6) and 32% mobile phase B (acetonitrile). The injection volume was 10 μl. Standard curves for Reb A (99%) and Reb D (97%) were generated as the external standard method for quantification.

## Results and Discussion

### Phylogeny Analysis and Rapid Screening of Glycosyltransferase for Rebaudioside D Synthesis

To discover a novel enzyme for Reb D synthesis, phylogenetic analysis of protein sequences was performed for mining functional enzymes, in which the protein sequence of UGT91D2 from *S. Rebaudiana* was used as a template. There were 4,510 sequences with homology between 30 and 90% and the query covered greater than 40 sequences screened from the NCBI BLAST (http://blast.ncbi.nlm.nih.gov/Blast.cgi). Enzymes from *Siraitia grosvenorii* with similar glycosylation mechanisms were not recorded in the NCBI database, which was manually added for phylogenetic analysis (Itkin et al., 2016). In total, there were 4,520 sequences available for phylogenetic analysis. The glycosyltransferase sequences were aligned with MAFFT v7.475 (Katoh and Standley, 2014). The phylogenetic trees were constructed with approximately maximum-likelihood methods by FastTree (Price et al., 2009).

**FIGURE 1:**
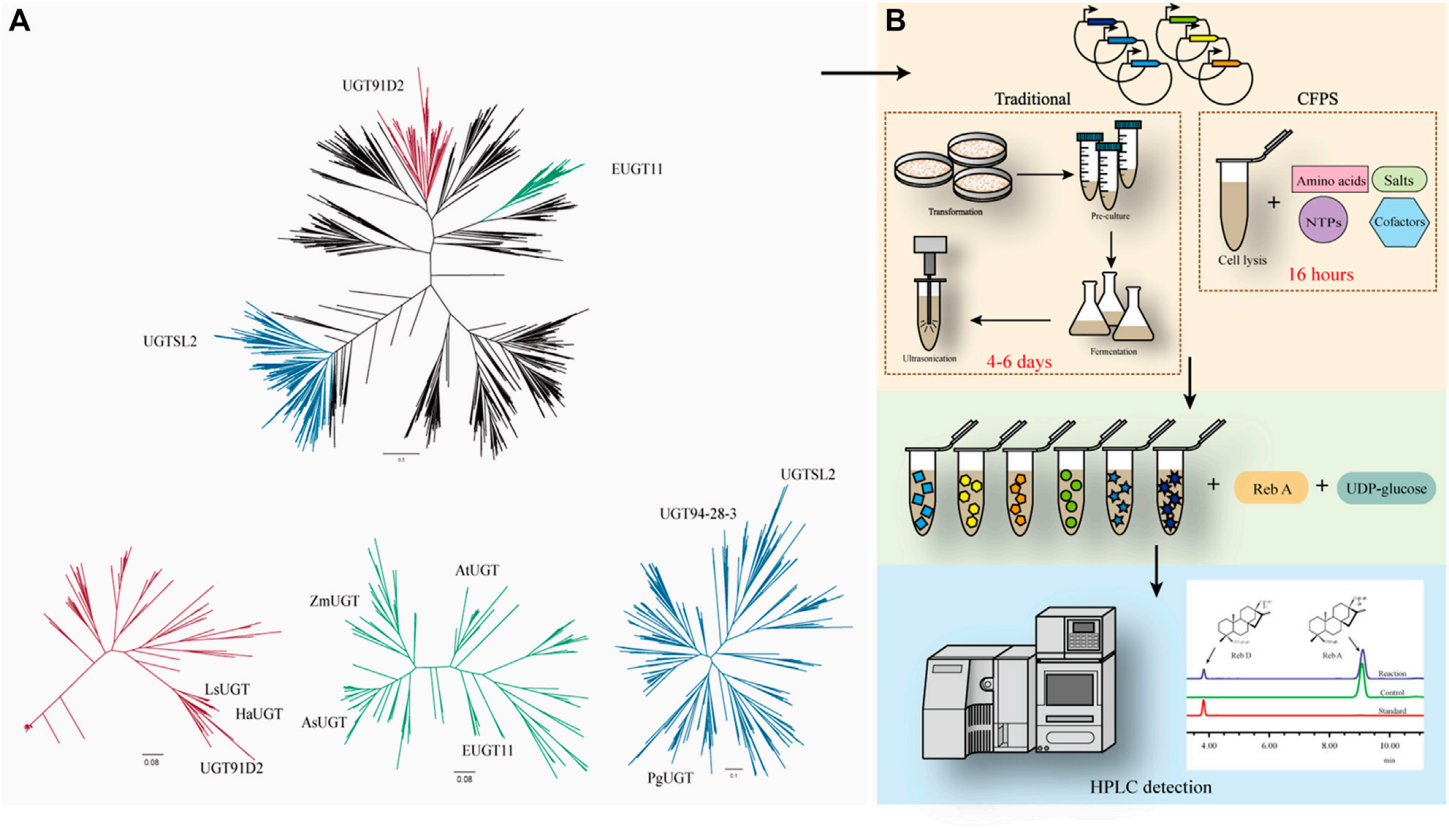
**(A)** Phylogenetic analysis of UGTs with UGT91D2 as the reference. UGT91D2 is located in the red sub-branch, EUGT11 is located in the green sub-branch, and UGTSL2 is located in the blue sub-branch. **(B)** Framework for the rapid screening of enzymes with a cell-free protein synthesis system.

In previous studies, there were three enzymes, namely, UGT91D2, EUGT11, and UGTSL2 capable of catalyzing Reb A to Reb D. The similarity between EUGT11 and UGT91D2 was 41.99%, while UGTSL2 shared 32.73% of its identity with UGT91D2. Despite the fact that these enzymes had the same catalytic capacity, they were separated into three phylogenetic branches (Figure 1A). UGTs in these three main sub-branches potentially have the glycosylation capacity of Reb A. Eight UGTs were chosen for further examination to find new enzymes for Reb D production. Among these sequences, LsUGT and HaUGT were chosen from the sub-branch of UGT91D2, whereas AsUGT, AtUGT, and ZmUGT were selected from the sub-branch of EUGT11. In the sub-branch of UGTSL2, PgUGT and UGT94-28-3 were picked. In addition, CsUGT was chosen from the other sub-branches. The information of these eight GTs is summarized in Table 1.

**TABLE 1:** Information of the glycosyltransferases selected by phylogenetic analysis.

Enzyme	Identity with UGT91D2 (%)	Genebank number	Number of amino acids	Molecular weight (kDa)	Isoelectric point	Source of organism	Phylogenetic sites
AsUGT	36.07	AZQ26909.1	486	52.92	5.81	*Avena strigosa*	EUGT11-subbranch
AtUGT	39.44	XP_020148974.1	454	49.22	6.34	*Aegilops tauschii subsp. strangulata*	EUGT11-subbranch
ZmUGT	39.09	NP_001150595.1	470	51.29	7.26	*Zea mays*	EUGT11-subbranch
LsUGT	65.04	XP_023735445.1	474	53.31	5.29	*Lactuca sativa*	UGT91D2-subbranch
HaUGT	66.67	XP_022009959.1	493	55.36	5.34	*Helianthus annuus*	UGT91D2-subbranch
CsUGT	38.46	ALO19883.1	469	52.23	6.41	*Camellia sinensis*	Other sub-branch
PgUGT	34.40	A0A0A6ZFY4.1	442	49.13	5.62	*Panax ginseng*	UGTSL2-subbranch
UGT94-28-3	34.00	Itkin et al. (2016)	473	52.83	5.78	*Siraitia grosvenorii*	UGTSL2-subbranch

To verify the catalytic properties of the enzymes chosen from the phylogeny tree, the DNA sequences of the enzymes were synthesized and inserted in the vectors for expression. The traditional approaches to determine the catalytic ability of the proteins take at least 4 days from the acquisition of the target DNA sequence to the determination of its properties if *E. coli* is used as the host generally. The cell-free protein synthesis system (CFPS) of *E. coli* has successfully synthesized a variety of proteins (Dudley et al., 2020; Karim et al., 2020). It takes only 16 h to obtain free proteins in an optimized CFPS after adding the vectors, significantly reducing the validation time (Figure 1B).

As a positive control for the feasibility of the CFPS, the gene of EUGT11 was inserted into the vector and added into the system and the other eight selected sequences. After 16 h of protein synthesis, the supernatant from the CFPS was added to the reaction system with Reb A and UDP-glucose as substrates. According to the HPLC results of SGs, the glycosylated product of Reb A catalyzed by EUGT11 had the same HPLC retention time as Reb D (Lin et al., 2020; Wang et al., 2020). In addition, PgUGT exhibited glycosylation ability on Reb A as EUGT11 among eight UGTs, which indicated that PgUGT would be a novel enzyme suitable for Reb D synthesis.

### Heterologous Expression and Purification of Glycosyltransferase From *P. ginseng*

**FIGURE 2:**
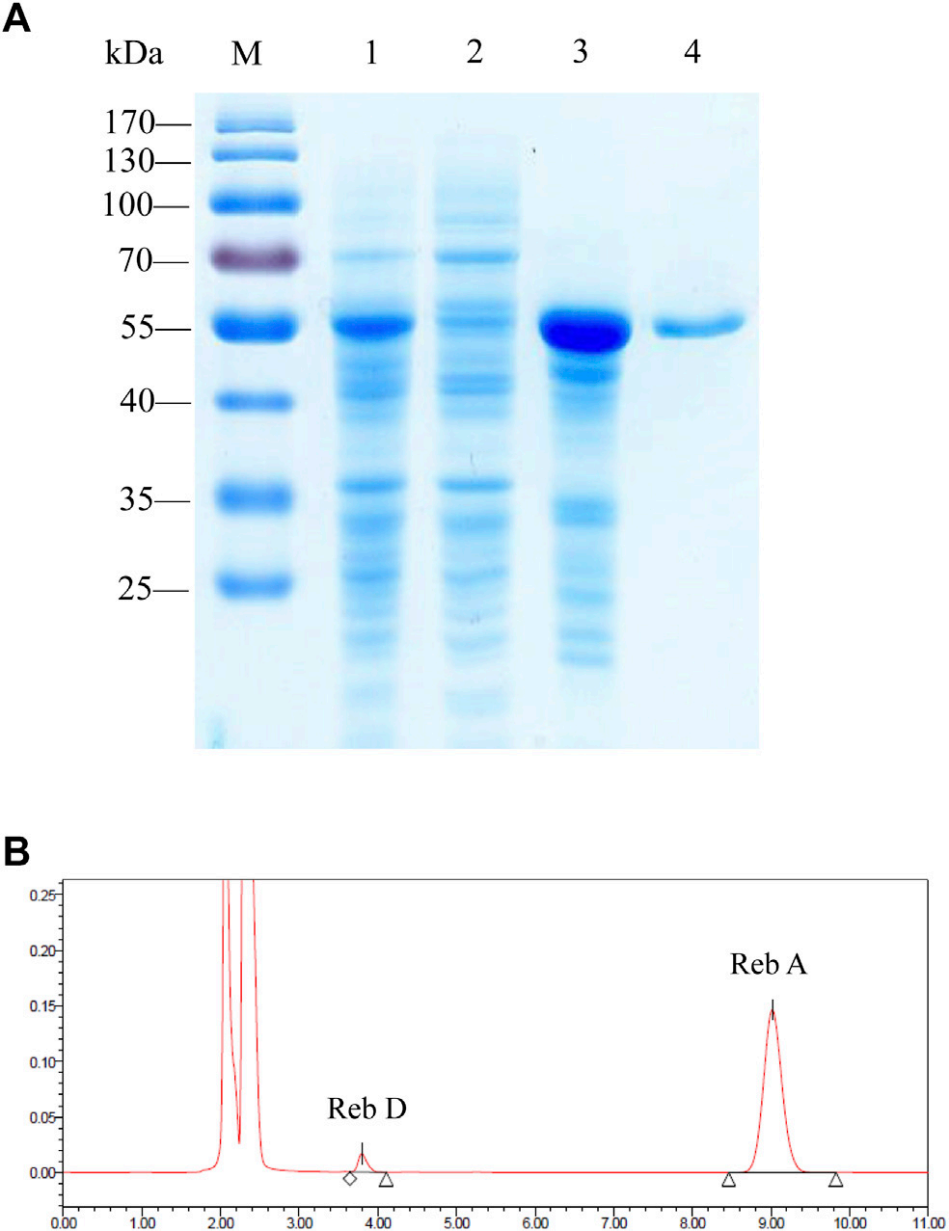
**(A)** SDS-PAGE analysis of the proteins during the purification process of PgUGT. Lane M, protein molecular weight standards; lane 1, cell lysate; lane 2, supernatant; lane 3, pellet; and lane 4, purified enzyme. **(B)** HPLC detection of the application of PgUGT on Reb D synthesis.

After the rapid screening of eight enzymes with the CFPS, the plasmid containing PgUGT was expressed in *E. coli* BL21 (DE3) for further characterization. After induction with 0.5 mM ITPG at 16°C for 24 h, the fermented cells were collected and disrupted by ultrasonication. The ginseng-derived UGT forms inclusion bodies when expressed in *E. coli,* and active enzymes were obtained by purifying soluble proteins (Figure 2A). The PgUGT protein carries a His-tag, and an enterokinase cleavage site at the N-terminus was purified *via* nickel column affinity chromatography. The purified enzyme showed a single band on the SDS-PAGE gel with a theoretical molecular mass of 54.9 kDa.

The catalytic ability of purified protein was verified by reactions using Reb A as the substrate. The HPLC results confirmed that PgUGT expressed in *E. coli* could synthesize Reb D from Reb A (Figure 2B).

### Biochemical Characterization of PgUGT

**FIGURE 3:**
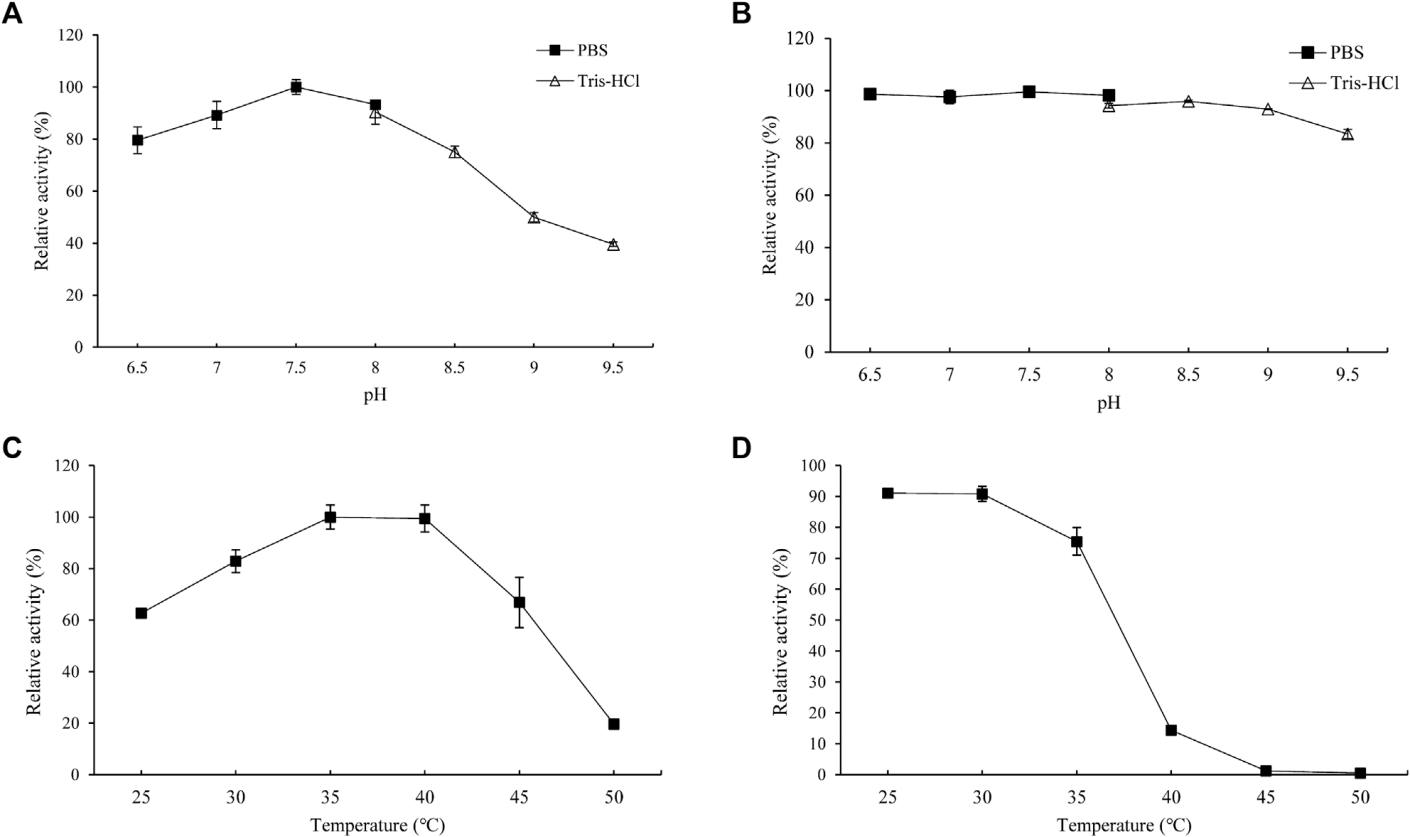
Optimal pH **(A)**, pH stability **(B)**, optimal temperature **(C)**, and thermal stability **(D)** of PgUGT. Error bars represent standard deviations, and three replicates were performed.

The effects of pH on the enzyme activity and stability of PgUGT were determined (Figures 3A,B). The optimum pH of PgUGT was 7.5. In a previous study, the optimal pH of EUGT11 expressed in *E. coli* was 8.5 (Wang et al., 2020). At pH 6.5–8.0, it exhibited more than 80% of total activity. The stability of PgUGT was high in this range of pH as well. However, the activity reduced greatly at a pH value of more than 8.5. The residual activity remained more than 92% when incubated in two buffers at pH 6.5–9.0 for 24 h. Therefore, the optimal reaction pH and pH stability revealed that PgUGT was a neutral enzyme.

The effect of temperature on PgUGT activity was explored at various temperatures ranging from 30–50°C. PgUGT exhibited maximum enzyme activity at 35°C, but the relative activity was similar at 40°C (Figure 3C). The activity showed a significant decrease over 40°C. The optimal temperature of EUGT11 expressed in *E. coli* was 35°C (Wang et al., 2020). A previous research study has shown that the optimum reaction temperature of plant-derived UGTs expressed in *E. coli* is usually between 30 and 35°C (Gachon et al., 2005; Louveau and Osbourn, 2019). As shown in Figure 2D, the thermostability of PgUGT remained 90% at 25–30 °C after 30 min of incubation. When the temperature increased, the thermostability decreased. Only 14% of enzyme activity remained after 30 min of incubation at 40°C.

For industrial application, thermostability and enzyme activity play important roles in the feasibility of enzymes (Xu et al., 2020). A higher application temperature in biocatalyst production ensures reduction in microbial contamination, better solubility, and often a more favorable equilibrium position (Bommarius and Paye, 2013). In a previous study, the optimal temperature for Reb A synthesis by the whole-cell biocatalyst was 50°C, which was favorable for industrial application (Chen et al., 2021). Considering the possibility of combining PgUGT and UGT76G1 to utilize St as a substrate for Reb D synthesis, the thermostability of PgUGT should be improved.

### Structural Modeling of PgUGT

**FIGURE 4:**
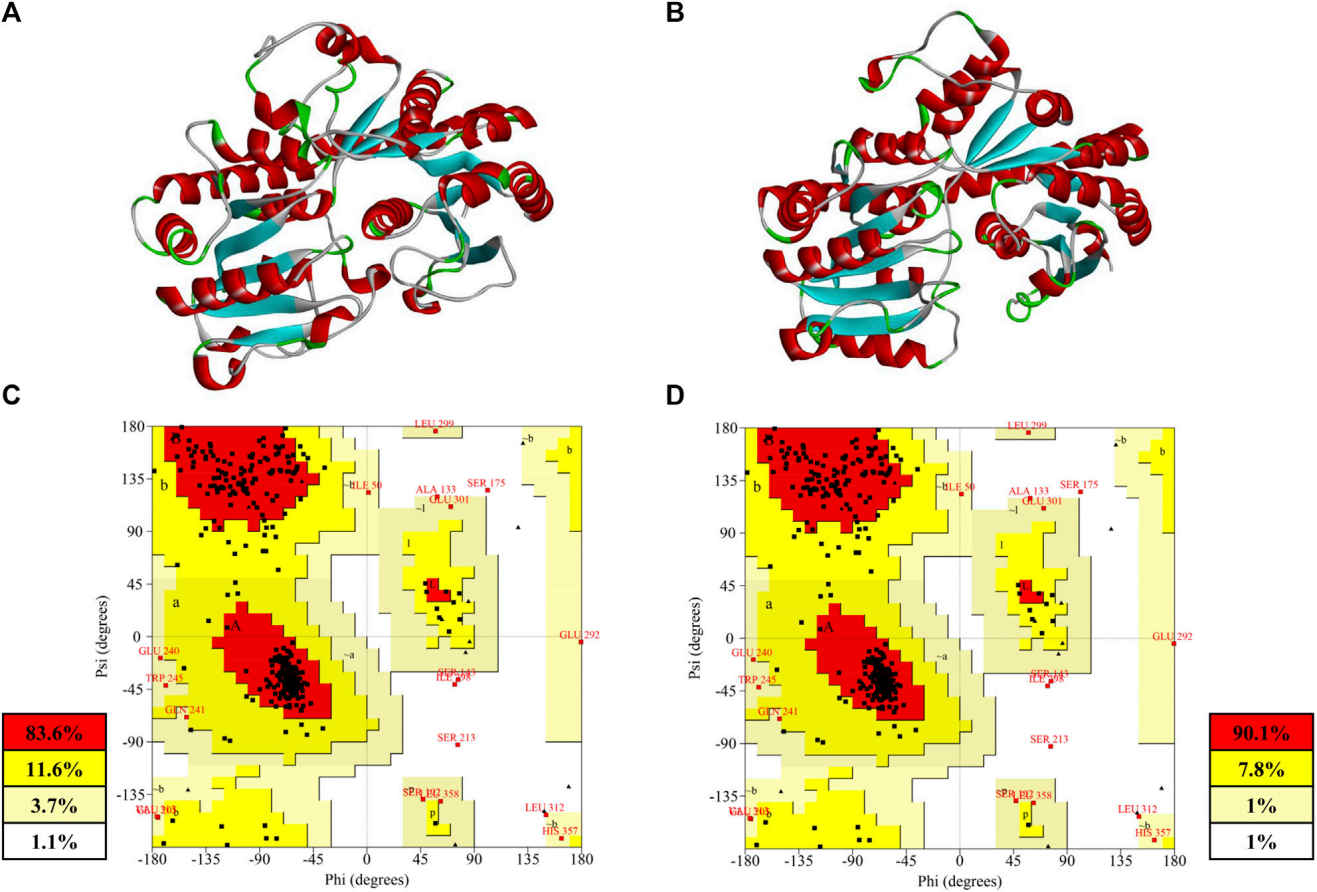
**(A)** Homology model built by Discovery Studio; **(B)** deep-learning–based model built by RoseTTAFold; **(C)** Ramachandran plot of the homology model; **(D)** Ramachandran plot of deep-learning-based model.

The crystal structure of mesophilic and thermophilic proteins revealed the relationship between protein configuration and thermostability (Haney et al., 1999). However, there was no crystal structure for PgUGT. Therefore, two structures of PgUGT were predicted to improve the thermostability and activity of PgUGT by a semi-rational design (Figures 4A,B). One was modeled *via* homology modeling by Discovery Studio 2020 based on the structure of 2vce, 5u6m, 5u6n, 5v2k, 6inf, 6ing, 6kvi, and 6o86. The alignment among the sequences is shown in Supplementary Figure S1. Homology modeling based on crystal structures with high sequence similarity is the most common approach. The lower the sequence similarity, the less accurate the model will be, which is the bottleneck of homology modeling (Muhammed and Aki-Yalcin, 2019). Recently, machine learning–based modeling was favored by researchers, which complements the deficiency of homology modeling with high accuracy (Baek et al., 2021). Another 3D-structure of PgUGT was modeled through the deep learning modeling method RoseTTAFold (https://robetta.bakerlab.org/).

Both of the modeled structures of PgUGT consisted of two Rossmann-like domains at the N-terminal and C-terminal, which was a typical characteristic of GT-B fold glycosyltransferase (Lee et al., 2019; Zhang et al., 2021). The accuracy of the structure prediction of two structures was further confirmed by the Ramachandran analysis with SAVES v6.0 (https://saves.mbi.ucla.edu) (Figures 4C,D). Generally, a good quality model would be expected to have more than 90% residues in the most favored regions, and a reliable model should have more than 80% (Laskowski et al., 1993). As shown in Figures 4C,D, 90.1% of the residues in the model predicted by RoseTTAFold were found in the most favored regions, while there were 83.6% residues in the homology model in the same regions. Both of the models were reliable for subsequent analysis and a better quality was shown in the model predicted by deep learning.

### Semi-Rational Design of Potential Thermostable Mutants

In previous studies, the semi-rational design by using computational tools in protein engineering was a common strategy. Improving thermostability may decrease the enzyme activity because it could change the flexibility of the structure (Xu et al., 2020). But, there are cases in which the thermostability was improved and the enzyme activity was increased by using FireProt (Cheng et al., 2020; Xia et al., 2021). Protein analysis tools such as Rate4Site, FoldX, and Rosetta design were assembled to offer a reliable design of stable multiple-point mutants in FireProt (Musil et al., 2017).

In this study, both of the predicted structures of PgUGT were analyzed by the FireProt server for calculation. As a result, 62 mutations were predicted based on the homology model and 45 mutations were shown based on the RosseTTAFold model. After cross matching the mutants, 16 site-directed mutants were selected. These mutants were shown in the results of both the models after excluding those with elevated energy (Table 2). Considering that all of the mutants were predicted based on modeled structures, to filter the mutants which would cause the inactivation of PgUGT, the single mutants were characterized.

**TABLE 2:** Information of the mutations selected for characterization.

Mutation	Conserved	Homology model ΔΔG (kcal·mol^−1^)		RoseTTAFold model ΔΔG (kcal·mol^−1^)	
		FoldX	Rosetta	FoldX	Rosetta
A11L	Y^[^ ^a^ ^]^	−3.20	-^[^ ^c^ ^]^	−1.90	—
V38I	Y	−1.0	—	−0.23	—
F39Y	Y	−0.47	—	−0.66	—
D54L	N^[^ ^b^ ^]^	−0.04	—	−3.26	−3.61
S55P	N	−1.13	−2.43	−0.1	—
S58G	N	−2.12	—	−3.2	−2.77
N109K	N	−0.62	—	−2.98	−2.79
S120P	N	−0.3	—	−0.46	—
G147W	N	−1.17	−2.35	−1.9	−7.13
A250E	N	−0.58	—	−0.7	—
I279L	N	−0.9	—	−0.35	—
L290F	Y	−0.33	—	−0.95	—
V304L	N	−0.8	—	−1.11	−9.27
Q305E	N	−0.45	—	−0.64	—
T329I	Y	−1.78	—	−0.98	—
R356M	Y	−2.92	—	−0.55	—

a Residue on the given position is conserved through protein evolution.

b Residue on the given position is not conserved through protein evolution.

c Data are not provided by FireProt.

**FIGURE 5:**
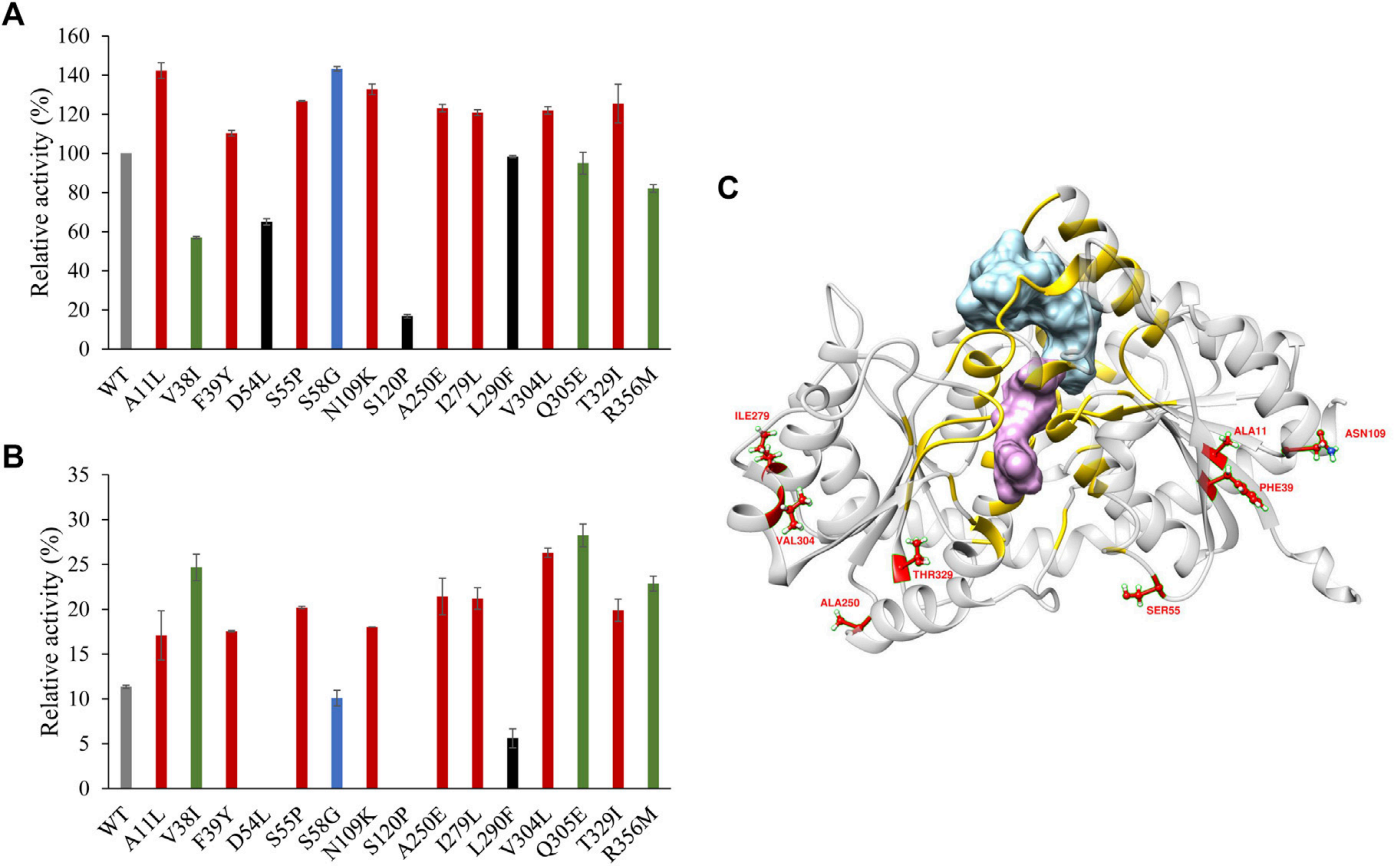
**(A)** Enzyme activity of PgUGT variants. **(B)** Thermostability of PgUGT variants. Red, variants with both higher enzyme activity and better thermostability; green, variants with only better thermostability; blue, variants with only higher enzyme activity; black, variants with both reduced enzyme activity and worse thermostability. **(C)** PgUGT model docked with Reb A and UDP-glucose. Blue, Reb A; purple, UDP-glucose; yellow, residues around Reb A and UDP-glucoside within 6 Å. Error bars represent standard deviations, and three replicates were performed.

Sixteen variants were constructed and expressed in *E. coli* BL21 (DE3). To determine the thermostability of the variants, all the purified proteins were incubated at 40°C for 30 min. The activity of the enzymes without heat treatment and the residual activity after incubation were determined under optimal reaction conditions. Among the 16 site-directed mutants, G147W was devoid of enzyme activity. Nine of them showed more than 10% higher enzyme activity than the WT. At the same time, 11 mutants had better thermostability than the WT. Consequently, the variants A11L, F39Y, S55P, N109K, A250E, I279L, V304L, and T329I had both of the aforementioned enhancements, which could be combined for further improvement (Figures 5A,B).

With the RoseTTAFold Model predicted with the WT sequence, the structures of Reb A and UDP-glucose were docked into PgUGT by Ledock (Zhang and Zhao, 2016). The docking conformations were evaluated based on the positions of ligands and the scores provided by Ledock. As shown in Figure 5C, the residues located within the distance of 6 Å around Reb A and UDP-glucose were yellow, which might be the key residues of the active pocket. However, all of the eight mutations were out of that area, suggesting that these residues may influence the substrate channel gating functions (Chen J. et al., 2020).

### Characterization of Combination Mutation Mut8

Based on the characterization of site-directed mutants, a combination mutation containing the eight mutations (A11L/F39Y/S55P/N109K/A250E/I279L/V304L/T329I, Mut8) was constructed and analyzed. After fermentation and purification, the enzyme activity of Mut8 was determined after adjusting it to the same concentration of the WT at 35°C. The specific activity of WT PgUGT was 1,290.5 U·mg^−1^, whereas the activity of the Mut8 reached 4122.5 U·mg^−1^, showing approximately a 3.2-fold increase (Table 3). The kinetics of the WT and Mut8 was evaluated by the Michaelis–Menten equation (Supplementary Figure S2). Both of them were incubated with different concentrations of the substrates. When compared to EUGT11 and UGTSL2, the WT of PgUGT and Mut8 had a similar affinity for Reb D to EUGT11 and was higher than UGTSL2 (Chen L. et al., 2020; Lin et al., 2020). Although Mut8 showed lower affinity for Reb A than the WT, the *k*_cat_ value of Mut8 indicated a higher catalytic rate. Consequently, the kinetic parameters of WT and Mut8 showed that the catalytic efficiency of Mut8 (*k*_cat_/*K*_m_ = 394.3 min^−1^mM^−1^) was 2.14-fold higher than that of the WT (*k*_cat_/*K*_m_ = 184.2 min^−1^mM^−1^).

**TABLE 3:** Specific activity and kinetic parameters of WT PgUGT and Mut8.

Enzyme	Specific activity (U mg^−1^)	*k*_cat_ (min^−1^)	*K*_m_ (mM)	*k*_cat_/*K*_m_ (min^−1^ mM^−1^)<
WT	1290.5 ± 52.0	22.1 ± 1.9	0.12 ± 0.0047	184.2
Mut8	4,122.5 ± 73.2	82.8 ± 4.7	0.21 ± 0.0046	394.3

**FIGURE 6:**
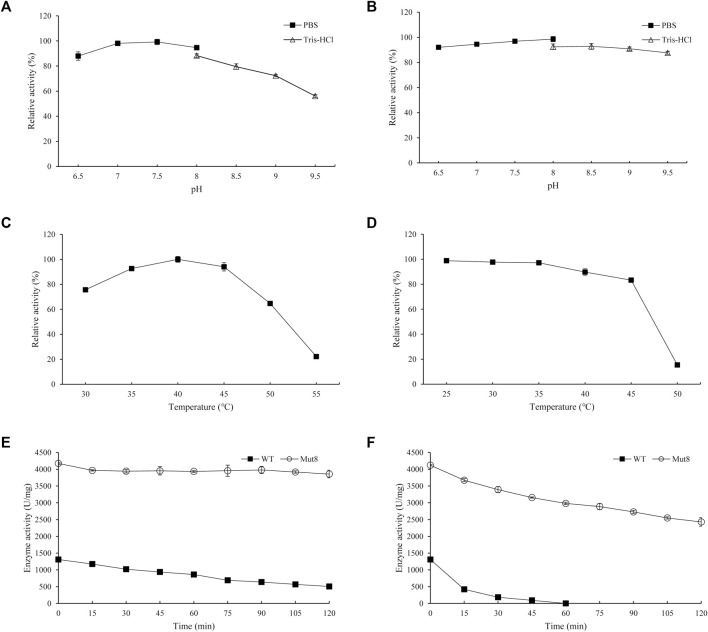
**(A)** Optimal pH of Mut8; **(B)** pH stability of Mut8; **(C)** optimal temperature of Mut8; **(D)** thermal stability of Mut8; **(E)** thermostability of WT and Mut8 incubated at 35°C; **(F)** thermostability of WT and Mut8 incubated at 40°C. Error bars represent standard deviations, and three replicates were performed.

Furthermore, the biochemical characteristic of Mut8 was determined. The optimal pH of Mut8 was consistent with that of the WT for Reb D synthesis (Figures 6A,B). Similarly, the Mut8 maintained over 92% activity after 24 h of incubation in buffers, of which the pH ranged from 6.5 to 9.0. A significant improvement was found in the curves of optimum temperature and thermostability (Figures 6C,D). The optimal temperature of enzyme activity increased from 35 to 40°C. The residual activity of Mut8 remained 83.3 and 15.4% after incubation at 45 and 50°C for 30 min, respectively, while WT was almost completely inactivated. Compared with the activity without heat treatment, the enzyme activity of the WT was reduced to 38.5% after incubation at 35°C for 2 h, while the residual enzyme activity of Mut8 remained over 93% (Figure 6E). Meanwhile, 59.0% of Mut8 activity was retained after incubation at 40°C for 2 h even though the WT was inactive within 1 h (Figure 6F). These results indicated that Mut8 was a mutant with significant improvement in both enzyme activity and thermostability compared to those in WT, which made it more suitable for industrial application.

## Conclusion

In this study, PgUGT from *P. ginseng* was characterized for Reb D synthesis. The optimal pH and temperature of the purified PgUGT were pH 7.5 and 35°C, respectively. Although the relative enzyme activity of PgUGT was stable in the pH range of 6.5–9.0, its thermostability decreased significantly over 35°C. Based on the two kinds of structure modeling and FireProt, 16 site-directed variants were constructed and validated. Eight of them showed improvement in both enzyme activity and thermostability. Furthermore, a combined mutant Mut8 containing eight mutations was characterized, which had a 3.2-fold increase in the enzyme activity and significant enhancement on thermostability.

To the best of our knowledge, this is the first report wherein UGT from *P. ginseng* was found to act as catalysts in the production of Reb D from Reb A. In addition, the mutant Mut8 could be a great potential enzyme for the industrial production of Reb D and other steviol glycosides.

## Data Availability

The original contributions presented in the study are included in the article/Supplementary Material, further inquiries can be directed to the corresponding authors.
