# Supplementary material for: Improving Thermostability and Catalytic Activity of Glycosyltransferase From Panax ginseng by Semi-Rational Design for Rebaudioside D Synthesis
Source: Front Bioeng Biotechnol. 2022 Apr 27;10:884898. doi: 10.3389/fbioe.2022.884898 (PMC9092651; doi:10.3389/fbioe.2022.884898)
Supplement: Supplementary file 1 [file Table1.docx]

**Frontiers in Bioengineering and Biotechnology**

**Supporting Information for**

**Improving thermostability and catalytic activity of glycosyltransferase from *Panax ginseng* by semi-rational design for rebaudioside D synthesis**

Meiqi Chen^a^ · Fangwei Song^a^ · Yuxi Qin^a^ · Shuangyan Han^a^ · Yijian Rao^b^· Shuli Liang^a,^ * · Ying Lin ^a,^ *

^a^ Guangdong Key Laboratory of Fermentation and Enzyme Engineering, School of Biology and Biological Engineering, South China University of Technology, Guangzhou, China

^b^ Key Laboratory of Carbohydrate Chemistry and Biotechnology, Ministry of Education, School of Biotechnology, Jiangnan University, Wuxi 214122, PR China

* Corresponding author.

**E-mail addresses:** **[shuli@scut.edu.cn](mailto:shuli@scut.edu.cn) (Shuli Liang);** **[feylin@scut.edu.cn](mailto:feylin@scut.edu.cn) (Ying Lin)**

**Table S1**. Site-directed mutation primers used in this study

| Primer | Sequence |
| --- | --- |
| A11L-F | AATCAGTATACTGTTGCTACCATTTTTAGC |
| A11L-R | CAGTATACTGATTCTACCAT |
| V38I-F | AAATTGCAATATTTTCCTCTGTTCTACCCC |
| V38I-R | AATATTGCAATTTCTTTTCG |
| F39Y-F | TGCAATGTTTATCTCTGTTCTACCCCAATC |
| F39Y-R | ATAAACATTGCAATTTCT |
| D54L-F | CAAGGATAAGCTGTCCTCTGCTTCTATAAAA |
| D54L-R | CAGCTTATCCTTGATGGAGCT |
| S55P-F | AGGATAAGGATCCGTCTGCTTCTATAAAAC |
| S55P-R | CGGATCCTTATCCTTGATGG |
| S58G-F | GGATTCCTCTGCTGGTATAAAACTAGTTGAG |
| S58G-R | ACCAGCAGAGGAATCCTTAT |
| N109K-F | CTTAAAACCTTAAAACCCGATTTGCTTATTT |
| N109K-R | TTTTAAGGTTTTAAGGATTTC |
| S120P-F | TTCAATCCCCCGTGGGCACCGGAGATCGCT |
| S120P-R | CGGGGGATTGAAATCATAAA |
| G147W-F | GCTCTTCCATTTGGCTACATGCTTTCAAAAA |
| G147W-R | CCAAATGGAAGAGCTGGCT |
| A250E-F | GACAAAAGGGAAGAATCTACAGTGGTGTTT |
| A250E-R | TTCCCTTTTGTCAAGCCAGT |
| I279L-F | TGGGCTAGAGCTGAGCACGGTTAATTTCAT |
| I279L-R | CAGCTCTAGCCCAATTGCT |
| L290F-F | GCTGTGAGATTTATTGAAGGAGAGAAAAA |
| L290F-R | AAATCTCACAGCCCATATGA |
| V304L-F | ACCAGAGGGGTTTCTGCAAAGGGTAGGAGAC |
| V304L-R | CAGAAACCCCTCTGGTAAAA |
| Q305E-F | GGGTTTGTTGAAAGGGTAGGAGACAGAGGAT |
| Q305E-R | TTCAACAAACCCCTCTGGTA |
| T329I-F | CATTCAAGCATTGGTGGGTTTGTGAGCCAT |
| T329I-R | AATGCTTGAATGTCCTAAAA |
| R356M-F | TGCCATGGCCATGCATCTTGATCAGCCTTT |
| R356M-R | CATGGCCATGGCAATTACTG |
| S58G/S55P-F | TCCGTCTGCTGGTATAAAACTAGTTGAGCTT |
| S58G/S55P-R | ACCAGCAGACGGATCCTTA |

**Figure S1**. Sequence alignment among PgUGT, 5v2k, 5u6n, 2vce, 6kvi, 6o86, 6ing and 6inf.


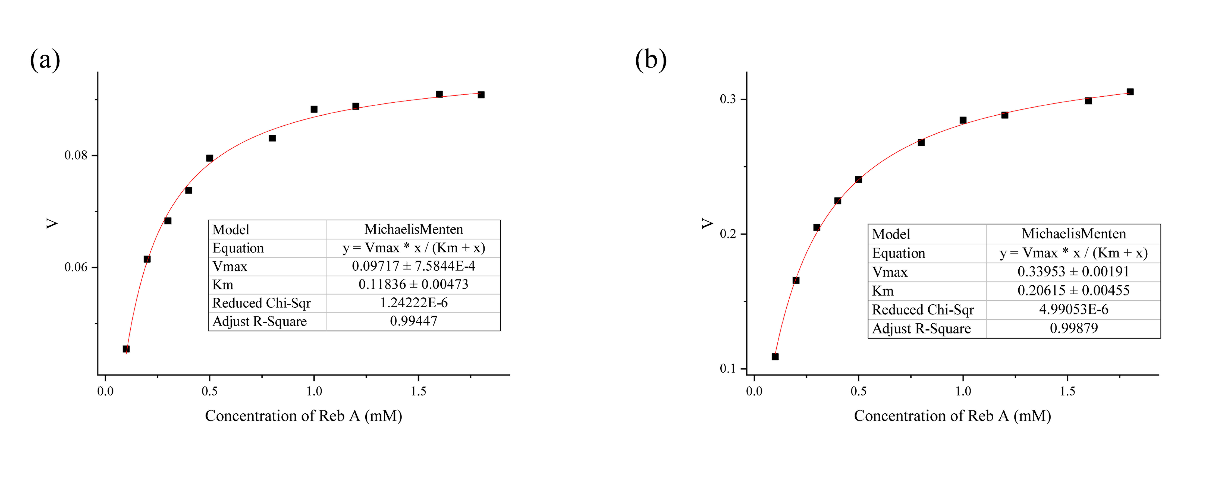


**Figure S2.** Michaelis-Menten fitted curves of WT (a) and Mut8 (b).
